# Genetic Researchers’ Use of and Interest in Research With Diverse Ancestral Groups

**DOI:** 10.1001/jamanetworkopen.2024.6805

**Published:** 2024-04-16

**Authors:** Kaitlyn Jaffe, Amanda K. Greene, Luyun Chen, Kerry A. Ryan, Chris Krenz, J. Scott Roberts, Brian J. Zikmund-Fisher, Amy L. McGuire, J. Denard Thomas, Erica E. Marsh, Kayte Spector-Bagdady

**Affiliations:** 1 Department of Health Promotion and Policy, University of Massachusetts, Amherst; 2 Center for Bioethics and Social Sciences in Medicine, University of Michigan Medical School, Ann Arbor; 3 Department of Obstetrics and Gynecology, University of Michigan Medical School, Ann Arbor; 4 Department of Health Behavior and Health Education, University of Michigan School of Public Health, Ann Arbor; 5 Department of Internal Medicine, University of Michigan Medical School, Ann Arbor; 6 Center for Medical Ethics and Health Policy, Baylor College of Medicine, Houston, Texas; 7 Michigan Institute for Clinical and Health Research, University of Michigan, Ann Arbor

## Abstract

**Importance:**

Genetic researchers must have access to databases populated with data from diverse ancestral groups to ensure research is generalizable or targeted for historically excluded communities.

**Objective:**

To determine genetic researchers’ interest in doing research with diverse ancestral populations, which database stewards offer adequate samples, and additional facilitators for use of diverse ancestral data.

**Design, Setting, and Participants:**

This survey study was conducted from June to December 2022 and was part of an exploratory sequential mixed-methods project in which previous qualitative results informed survey design. Eligible participants included genetic researchers who held US academic affiliations and conducted research using human genetic databases.

**Exposure:**

Internet-administered survey to genetic research professionals.

**Main Outcomes and Measures:**

The survey assessed respondents’ experience and interest in research with diverse ancestral data, perceptions of adequacy of diverse data across database stewards (ie, private, government, or consortia), and identified facilitators for encouraging use of diverse ancestral data. Descriptive statistics, χ^2^ tests, and *z* tests were used to describe respondents’ perspectives and experiences.

**Results:**

A total of 294 researchers (171 men [58.5%]; 121 women [41.2%]) were included in the study, resulting in a response rate of 20.4%. Across seniority level, 109 respondents (37.1%) were senior researchers, 85 (28.9%) were mid-level researchers, 71 (24.1%) were junior researchers, and 27 (9.2%) were trainees. Significantly more respondents worked with data from European ancestral populations (261 respondents [88.8%]) compared with any other ancestral population. Respondents who had not done research with Indigenous ancestral groups (210 respondents [71.4%]) were significantly more likely to report interest in doing so than not (121 respondents [41.2%] vs 89 respondents [30.3%]; *P* < .001). Respondents reported discrepancies in the adequacy of ancestral populations with significantly more reporting European samples as adequate across consortium (203 respondents [90.6%]), government (200 respondents [89.7%]), and private (42 respondents [80.8%]) databases, compared with any other ancestral population. There were no significant differences in reported adequacy of ancestral populations across database stewards. A majority of respondents without access to adequate diverse samples reported that increasing the ancestral diversity of existing databases (201 respondents [68.4%]) and increasing access to databases that are already diverse (166 respondents [56.5%]) would increase the likelihood of them using a more diverse sample.

**Conclusions and Relevance:**

In this survey study of US genetic researchers, respondents reported existing databases only provide adequate ancestral samples for European populations, despite their interest in other ancestral populations. These findings suggest there are specific gaps in access to and composition of genetic databases, highlighting the urgent need to boost diversity in research samples to improve inclusivity in genetic research practices.

## Key Points

**Question:**

Are genetic researchers interested in research with diverse ancestral groups, and how can data stewards encourage that use?

**Findings:**

In this survey study of 294 genetic researchers, significantly more respondents reported working with data from European ancestral populations than any other ancestral population, and European samples were more likely to be considered by researchers as adequate across data-steward type. Most researchers were interested in using more diverse ancestral populations and reported that increasing ancestral diversity of existing databases would enable such research.

**Meaning:**

These findings suggest that there are specific gaps in access to and composition of genetic databases, underscoring the need to boost diversity in existing research samples to improve inclusivity in genetic research practices.

## Teaser

This survey study assesses genetic researchers’ interest and experience with using diverse ancestral groups in their research.

## Introduction

In the era of precision medicine, genomic data are increasingly critical to refining and improving health care delivery.^1^ Through advances in translational research, genomic databanks can be used to associate genetic variation with both disease risk and treatment response (eg, pharmacogenomics).^2^

However, genomic databases generally lack demographic diversity across a number of variables. For example, the vast majority of genome-wide association studies (GWAS) are conducted with European ancestral populations,^3^ with more than 80% of individuals in GWAS being of European descent.^4^ In contrast, populations of African ancestral descent represent just 2% of the overall samples.^4,5^ This is particularly problematic given the high genetic diversity of populations of African ancestry and their data’s unique ability to contribute to advances in genomic medicine.^6^ For example, in a recent analysis of the National Human Genome Research Institute and European Bioinformatics Institute GWAS Catalogue,^7^ data from the 2.4% of participants of African ancestry contributed to 7% of associations of genetic variance with traits.

It is unclear whether the populations represented in genomic data also generally vary by type of database, including those managed by consortia (eg, 1000 Genomes Project or ENCODE), government (eg, UK Biobank, All of Us Research Hub, or the Database of Genotypes and Phenotypes), or private entities (eg, 23andMe, Ambry, or Ancestry DNA). There is also limited information regarding the ancestral representativeness of private databases; however, past work^7,8,9^ has indicated that these databases, like others, are largely comprised of European contributors. These disparities in the ancestral diversity of genomic data can impact the communities to which genomic research will generalize or can be targeted for and, consequently, impact health equity in genomic medicine.^10,11^

To address this homogeneity, government initiatives have sought to increase the representation of populations historically underrepresented in biomedical research. For example, as part of President Obama’s Precision Medicine Initiative, the $2 billion All of Us research program aims to recruit 1 million participants and build a cohort comprised of more than 45% individuals from self-identified racial and ethnic minority groups (which can be associated with ancestral background) and more than 75% from populations generally underrepresented in research.^12,13^ Other efforts to diversify genetic databases include the recent publishing of the Pangenome.^14^

Nevertheless, researchers remain limited in their access to demographically diverse genomic data. Our previous qualitative work^15^ with genetic researchers indicated a reinforcing, cyclical pattern: researchers know genetic databases will not be ancestrally diverse, and therefore do not prioritize such diversity in database selection. Others have normatively argued that racial and ethnic minority scientists are more likely to conduct research with racial and ethnic minority populations, so the lack of diversity in the scientific population compounds the lack of diversity in research sample populations.^16^

To support the effort toward increasing the generalizability of genomic research across diverse ancestries, we revisited these challenges through an online survey of academic US genetic researchers who use human genomic data from consortium, government, or private databases. The survey focused on researchers’ perceptions of genomic databases and views on genomic data sharing broadly. Here, we queried respondents’ experience and interest in research with diverse ancestral groups because there is potentially a difference between the relative representativeness of ancestries within a database and access to adequate data necessary for diverse genomic analytic methods.^16,17^ We also assessed the adequacy of representation of diverse ancestral populations by database steward type (ie, private, government, and consortia). Last, we explored facilitators to encourage their use.

## Methods

This survey study was deemed exempt from full review by the University of Michigan Medical School institutional review board because the study involved a survey and information was collected so that participant identity could not be readily obtained in accordance with the Common Rule. The study followed the American Association for Public Opinion Research (AAPOR) reporting guideline for survey studies. Potential respondents were eligible to complete the online Qualtrics survey if they had an affiliation with a US academic institution, published research utilizing a genetic database for human research, and had experience using consortia, government, and/or privately managed databases. The first page of the survey provided information regarding informed consent. A participant was deemed to have consented to the study if they proceeded to the next page.

### Recruitment

We used several different methods to recruit genetic researchers. Our first cohort was built through a systematic search of PubMed indexed articles that used human genetic databases. We focused on original research articles using human genetic data published between January 2017 and March 2021, in which the first or last author had a US academic affiliation. From this comprehensive search, we compiled a database of 1993 US genetic researchers, including their institutional contact information. We emailed all researchers in June 2022. We did a second wave of email recruitment to nonresponders in November 2022, including 2 follow-up emails in December 2022. Additionally, we sent a postcard with QR code linking to the survey to approximately one-third of the November recruits, but this approach had a negligible impact on response, so we followed-up with the remainder of our list by email only.

We additionally recruited, via a descriptive link to the survey, in the American Society of Human Genetics (ASHG) September 2022 and November 2022 email newsletters and by distributing postcards at both the ASHG and the American Society for Bioethics and Humanities annual conferences in October 2022. Due to a lack of member tracking at the professional organizational level, we were unable to establish the baseline of readers and attendees who met inclusion criteria through this method. All respondents were offered a $25 gift card for completion.

### Survey Design and Measures

We designed the survey instrument as part of an exploratory sequential mixed-methods project, in which previous qualitative results,^15^ also from US academic genetic researchers, informed the design and inclusion of key measures and the direction of this study. These novel measures assessed (1) general characteristics of the different kinds of genetic databases that respondents used (held by consortium, government, and private stewards [ie, the entities that manage and oversee many data resources]), (2) respondents’ perceptions of those different kinds of genetic databases, (3) perceived obstacles to their own research, and (4) general views on genetic data sharing (full survey available in the eAppendix in Supplement 1). We conducted 10 cognitive interviews to further refine the instrument.

Of key relevance to this analysis, we assessed which ancestral populations respondents had analyzed in their research, including African, American Indian or Alaskan Native, Arab or Middle Eastern, Asian, European, Hispanic or Latin American, mixed ancestries, Native Pacific or Pacific Islander, or other Indigenous populations. We also asked all respondents about their research interests related to different ancestral populations, the adequacy of database samples of ancestral populations across different data steward types (ie, consortium, government, and private) in their experience, and facilitators to conducting research with diverse ancestral populations. We also collected respondents’ own demographic characteristics, including self-identified gender, race, ethnicity, and career stage. Race and ethnicity categories for respondents included African American or Black, Asian, Hispanic or Latino, Indigenous, non-Hispanic White, multiracial (reported as mixed race on the survey), or none listed. Race and ethnicity information was collected because we wanted to understand whether respondent race and ethnicity might be associated with deciding to work with data from different ancestral groups. Survey measures are included in the eAppendix in Supplement 1.

While considering potential measures that would facilitate the incorporation of additional ancestral populations into their work, respondents were presented with several options derived from our initial qualitative analysis^14^ including (1) increasing the ancestral diversity of existing databases; (2) increasing access to ancestrally diverse databases; (3) additional methods development to support research in additional populations; (4) additional funding opportunities; (5) additional publication opportunities; (6) additional demographic data being included in the database; or (6) something else, with an option for open-text response.

### Statistical Analysis

We used descriptive statistics to describe respondents’ perspectives and experiences, and χ^2^ tests to understand whether respondent’s demographics or seniority were associated with those variables. We use *z* tests to compare the proportion of respondents who have experience and/or interest in research in different ancestral populations. We also assessed the adequacy of genetic databases by ancestral population and by type of data steward as perceived by respondents for their own research, respondents’ interest in working with different ancestral populations, and measures that would aid them in working with more diverse databases. We used SPSS version 28 (IBM) to complete all statistical analyses and to generate figures. All *P* values were 2-sided, and we considered *P* < .05 significant after a Bonferroni adjustment for multiple comparisons if warranted. Data analysis was conducted from April 2023 to March 2024.

## Results

Between June and December 2022, 1336 genetic research respondents (sampled via PubMed) opened our email, 373 opened the link to our survey, and 273 eligible respondents (20.4%) completed all questions (Table 1). An additional 21 participants fully completed our survey from our ASHG recruitment. In total, we had an analytic sample of 294 respondents (171 men [58.5%]; 121 women [41.2%]) (Table 2). The sample was majority non-Hispanic White (179 respondents [63.0%]), likely reflective of the field itself. Of all respondents, 4 (1.4%) identified as African American or Black, 70 (24.6%) as Asian, 12 (4.2%) as Hispanic or Latino, 9 (3.2%) as multiracial, 179 (63.0%) as non-Hispanic White, and 10 (3.4%) who did not list a race or ethnicity. Respondents ranged in seniority level, with 109 senior respondents (37.1%), 85 mid-level respondents (28.9%), 71 junior respondents (24.1%), and 27 trainee or student respondents (9.2%).

**Table 1.** Participant Recruitment

Recruitment process	Respondents, No./Total No. (%)
Authors identified via PubMed, No.	1993
Email successfully sent (did not bounce)	1911/1993 (95.8)
Emails opened	1336/1911 (69.9)
Surveys	
Survey link opened	373/1336 (27.9)
Survey incomplete	59/373 (15.8)
Surveys complete (finished)	273/373 (73.2)
Surveys complete but screened out	41/373 (11.0)
Surveys from authors identified via PubMed completed	273/1336 (20.4)
Surveys from American Society of Human Genetics completed, No.	21
Total surveys completed, No.	294

**Table 2.** Self-Reported Respondent Characteristics

Characteristic	Respondents, No. (%) (N = 294)
Gender	
Man	171 (58.5)
Woman	121 (41.2)
Neither of these describe me	2 (0.7%)
Race and ethnicity	
African American or Black	4 (1.4)
Asian	70 (24.6)
Hispanic or Latino^a^	12 (4.2)
Indigenous	0
None listed	10 (3.4)
Multiracial	9 (3.2)
Non-Hispanic White^a^	179 (63.0)
Seniority level	
Senior	109 (37.1)
Mid-level	85 (28.9)
Junior	71 (24.1)
Trainee or student	27 (9.2)

^a^ Respondents who identified as White and non-Hispanic or Latino were characterized as non-Hispanic White and those that identified as White and Hispanic or Latino were characterized as Hispanic or Latino for statistical purposes. There were no respondents who identified as Hispanic and Latino ethnicity and a race other than White.

### Experience and Interest in Research With Diverse Ancestral Data

**Figure 1.**
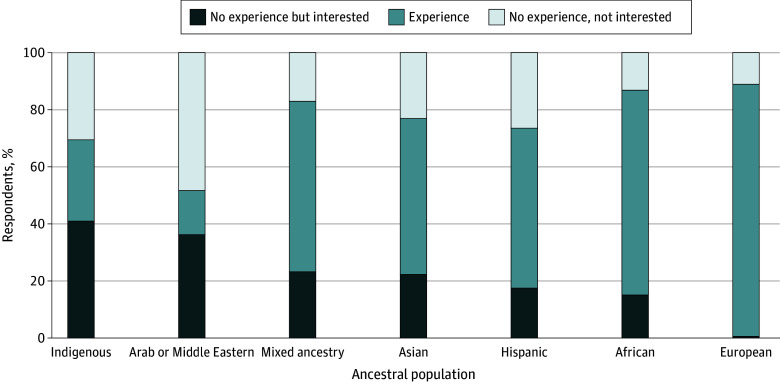
Experience and Interest in Using Data From Different Genetic Ancestral Groups

Respondents reported that most of their genetic research used data from participants of European ancestry (261 respondents [88.8%]). Significantly fewer respondents reported working with any other ancestral population as compared with European ancestry populations (Figure 1).

If a respondent reported that they had not used a specific ancestral population in the past, we queried whether they would be interested in so doing if they could. All descriptive results are displayed in Figure 1. A total of 210 respondents (71.4%) reported not having used samples from Indigenous ancestral groups (a variable we combined post hoc with Native Pacific or Pacific Islander and other Indigenous populations); these respondents were significantly more likely to report their interest in such research than not interested (121 respondents [41.2%] vs 89 respondents [30.3%]; *P* < .001). If respondents had not used samples from European ancestral groups in the past (33 respondents [11.2%]), they were significantly more likely to report not being interested in such research than being interested (31 respondents [10.5%] vs 2 respondents [0.7%]; *P* < .001) (Figure 1). Using χ^2^ tests, we found no significant association of the respondents’ own reported demographic characteristics (ie, race or ethnicity, gender, and seniority) with reported diversity of ancestral populations represented in their research.

### Adequacy and Use of Diverse Ancestral Populations Across Database Steward

**Figure 2.**
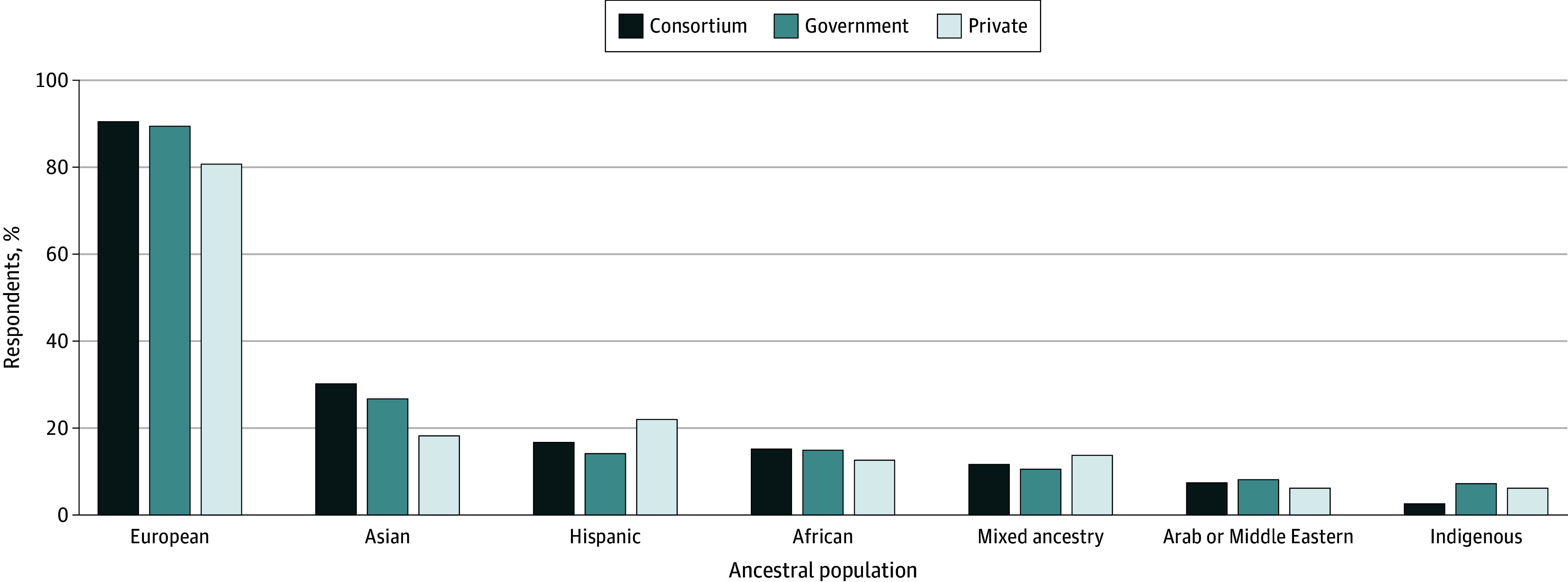
Respondents Reporting Ancestral Populations as Adequate by Ancestral Group and Data Steward Type

Respondents who reported research experience with a particular ancestral population and data steward were asked to self-evaluate the adequacy of the database steward’s sample for their respective research (ie, adequate, inadequate, or unsure). Respondents reported significant discrepancies in the adequacy of represented ancestral populations with significantly more reporting European samples to be adequate across consortium (203 respondents [90.6%]), government (200 respondents [89.7%]), and private (42 respondents [80.8%]) databases as compared with any other ancestral population. None of the other descriptive differences were statistically significant (Figure 2). In contrast with past reports focusing on the diversity of private databases, we found no significant differences in reported adequacy of ancestral populations across database steward.

### Facilitators for Use of Diverse Ancestral Populations

**Figure 3.**
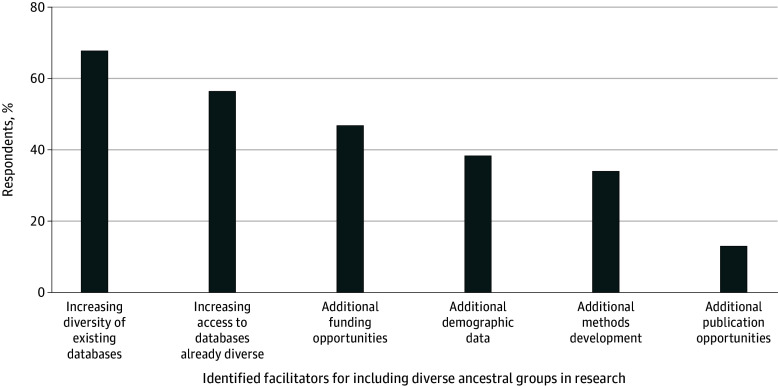
Facilitators for Use of Diverse Ancestral Populations in Genetic Research

For respondents who reported that they had not used a specific ancestral population in the past and indicated that they were interested in doing so (211 respondents [71.8%]), we asked what would increase the probability they could. A majority of respondents overall reported that increasing the ancestral diversity of existing databases (201 respondents [68.4%]) and increasing access to databases that are already ancestrally diverse (166 respondents [56.5%]) would increase the likelihood of them using more diverse ancestral populations. Others reported that additional funding opportunities (138 respondents [46.9%]), additional demographic data being included in the database (113 respondents [38.4%]), additional methods development (100 respondents [34.0%]), and additional publication opportunities (39 respondents [13.3%]) would increase the probability (Figure 3).

## Discussion

In this survey study, the majority of US academic genetic researchers who responded had experience or interest in using data from diverse ancestral groups. However, respondents reported that data sets across steward type were inadequate for their research across all ancestral groups but for European. Researchers thought increasing diversity of existing databases and access to existing diverse databases would be most likely to facilitate this research.

Researchers were significantly more likely to report working with European populations as compared with any other ancestral group, but the majority of respondents reported having worked with, or an interest in working with, all ancestral populations presented. Researchers who reported that they had not worked with diverse ancestral groups but were interested in doing so likely face different kinds of barriers related to the overall adequacy of population samples. For instance, the 0.7% of respondents who reported that they had no experience working with European populations but were interested were unlikely to be impacted by a lack of adequate samples, whereas respondents who reported that they had no experience working with African, Hispanic, and Indigenous populations likely were. In addition, while some have normatively argued that the demographic characteristics of researchers are associated with use of diverse data sets,^17^ our results showed no significant association of researcher demographics with the diversity of ancestral populations with whom they had done research. This finding highlights that a desire to work with diverse data may be a common, shared perspective in addition to the lack of access being a shared barrier.

Genetic researchers’ perceptions of the adequacy of ancestrally diverse samples reinforces previous findings demonstrating the lack of ancestral diversity across genomic databases.^3,5,10,18^ European populations were resoundingly the only population for whom the majority of researchers reported adequate access across respondents and database steward type. Notably, while government initiatives have dedicated substantial resources to increasing racial and ethnic diversity in genetic databases, few respondents reported databases from non-European ancestral populations to be adequate in government or (often government-funded) consortium databases. Crucially, this suggests that ensuring that there is some representation of diverse ancestral populations in a given genetic data set does not necessarily mean that the samples of those ancestral populations are adequate for researchers’ needs.

Focusing on the population of respondents who were interested in conducting research with an ancestral group with whom they had not worked, the primary reported facilitator was to increase the ancestral diversity of existing databases. In other words, respondents want existing databases to be more diverse—a finding we can contextualize within previous work,^15^ in which genetic researchers had an interest in using ancestrally diverse data, but this was not considered a priority when choosing a database. Thus, the research incentive structure is such that if data stewards themselves do not prioritize diversifying databases that researchers are already choosing, that prioritization will not happen at the researcher level. This might limit the impact of databases, such as All of Us, which exist independently, instead of integrating diverse data into more commonly used databases.

Respondents also reported increasing access to existing databases would facilitate more research with diverse ancestral populations. It may be that particular databases are prohibitively expensive or have greater restrictions on use and publishing, rendering them inaccessible. Some respondents also reported that increasing funding opportunities would help them conduct more research with diverse populations. Interpreted in the context of our previous qualitative findings,^15^ these funds would likely go toward supporting access to more costly data or providing the research infrastructure and personnel to support intensive data cleaning and analysis required to harmonize data from less represented ancestral populations across databases.^15^

### Limitations

This study has some limitations. First, we sampled a small and specific group of US research professionals. It is difficult to establish the total number of researchers using human genetic data with an academic affiliation in the US, so the extent to which these results are generalizable is limited. However, we did recruit respondents through multiple pathways, including the primary organization through which US genetic researchers are affiliated. Second, the potential for self-selection bias was a limitation. Genetic researchers who have stronger opinions about genetic databases may have been more likely to take the survey. That said, we did not disclose the content of the survey (eg, interest in data diversity) in the recruitment email. Third, there may have been some confusion between how we defined our 3 data stewards of interest (government, private, and consortium). To mitigate this impact, we did not use data from partial survey responses because several respondents reported that this confusion was why they did not complete the instrument. Fourth, we did not limit our analysis to only researchers who were specifically doing ancestral research. Fifth, respondents took the survey based on past research experience and their responses may not have captured the most recent government initiatives to emphasize and fund diverse genetic databases.

## Conclusions

In this survey study of US genetic researchers, respondents reported existing databases only provide adequate ancestral research data for European populations, despite their interest in other ancestral populations. Adequately representative genetic data from diverse ancestral populations are essential for health equity and for ensuring research outcomes are either directed toward or generalizable to diverse patient populations. Government-funded genetic databases in particular should be representative of the populations they serve and foster health innovations that benefit all communities. Although, it is important to note that diversity of ancestral groups represented in genomic databases is a necessary but not sufficient benchmark of the extent to which databases facilitate meaningful research with historically underrepresented groups. By understanding genetic researchers’ experiences working with data from different ancestral populations—as well as their perceptions of both sample adequacy and facilitators for doing genetic research with non-European populations—we can begin to identify specific gaps in the accessibility and composition of genetic databases and provide more robust support to researchers seeking to work with diverse ancestral populations.

## Notes

Supplement 1. **eAppendix.** Survey Instrument

Supplement 2.
Data Sharing Statement
