# Supplementary material for: Genetic Researchers’ Use of and Interest in Research With Diverse Ancestral Groups
Source: JAMA Netw Open. 2024 Apr 16;7(4):e246805. doi: 10.1001/jamanetworkopen.2024.6805 (PMC11022111; doi:10.1001/jamanetworkopen.2024.6805)
Supplement: Supplement 1. — eAppendix. Survey Instrument [file jamanetwopen-e246805-s001.pdf]

Supplemental Online Content

Jaffe K, Greene AK, Chen L, et al. Genetic researchers' use and interest in research with diverse ancestral groups. *JAMA Netw Open*. 2024;7(4):e246805.
doi:10.1001/jamanetworkopen.2024.6805

eAppendix. Survey Instrument

This supplemental material has been provided by the authors to give readers additional information about their work.

© 2024 Jaffe K et al. *JAMA Network Open.*

eAppendix. Survey Instrument

This is a survey about genetic researchers' experiences with, and opinions about, data sharing. You will be asked questions about your use of genetic databases, specifically those held by private entities, government entities, or through a consortium.

Although you may not directly benefit from being in this study, others may benefit from your participation. The goal of this project is to better facilitate genetic data sharing, access, and quality at the national level.

Your participation is completely voluntary. If you are not sure how to answer a question, just choose the response that most closely matches what you think. You may stop the survey at any time.

We expect this survey to take about 10-15 minutes for you to complete.

Do you have at least one U.S. academic affiliation? (Select ONE)

- o Yes
- o No

Have you published with human genetic data in the peer reviewed literature? (Select ONE)

- o Yes
- o No

What kinds of database stewards have you worked with now or in the past (including using summary statistics)? (Select ALL that apply)

- ☐ **CONSORTIUM** managed databases (that do not support individual researcher access but might otherwise be funded by or hosted by NIH)
- ☐ **GOVERNMENT** managed databases, including U.S. and international (e.g., UK Biobank, All of Us, dbGaP)
- ☐ **PRIVATELY** managed databases (e.g., 23andMe, Ambry, Color, Gene by Gene, Ancestry DNA)
- ☐ **OTHER** databases (e.g., academic, NGO), please specify below:

© 2024 Jaffe K et al. *JAMA Network Open.*

Which CONSORTIUM managed database(s) have you used? (Select ALL that apply)

- ☐ 1000 Genomes
- ☐ ENCODE
- ☐ WTCCC
- ☐ Others (please list)

Which GOVERNMENT managed databases have you used? (Select ALL that apply)

- ☐ All of Us
- ☐ dbGaP
- ☐ eMERGE
- ☐ TOPMed
- ☐ UK Biobank
- ☐ Others (please list):

Which PRIVATEY managed databases have you used? (Select ALL that apply)

- ☐ 23andMe
- ☐ Ambry Genetics
- ☐ Ancestry DNA
- ☐ Ariel Precision Medicine
- ☐ Color
- ☐ deCODE
- ☐ FoundationOne
- ☐ Gene by Gene
- ☐ Invitae
- ☐ Myriad
- ☐ Strata Oncology
- ☐ Others (please list):

What kind of human genetic data do you typically use? (Select ALL that apply)

- ☐ Epigenomic data
- ☐ Genome or Exome sequence data
- ☐ Single nucleotide polymorphism (SNP) array data
- ☐ Transcriptomic data
- ☐ Other molecular data produced by array-based technologies or high-throughput sequencing technologies
- ☐ Other (please explain):
- ☐ ⊗- *None of the above -*

What kind of data do you work with? (Select ALL that apply)

- ☐ Aggregate-level data
- ☐ Individual-level data
- ☐ Summary statistics
- ☐ Other (please explain):
- ☐ ⊗- *None of the above -*

© 2024 Jaffe K et al. *JAMA Network Open.*

Over the past 5 years, how has the extent of your use of CONSORTIUM databases changed in comparison to PRIVATE databases, if at all?(Select ONE)

- Using CONSORTIUM more
- No change
- Using PRIVATE more
- *Not Sure/ Cannot compare*

Over the past 5 years, how has the extent of your use of GOVERNMENT databases (with individual-level access) changed in comparison to CONSORTIUM databases, if at all? (Select ONE)

- Using GOVERNMENT more
- No change
- Using CONSORTIUM more
- *Not Sure/ Cannot compare*

Over the past 5 years, how has the extent of your use of PRIVATE databases changed in comparison to GOVERNMENT databases, if at all? (Select ONE)

- Using PRIVATE more
- No change
- Using GOVERNMENT more
- *Not Sure/ Cannot compare*

© 2024 Jaffe K et al. *JAMA Network Open.*

What kinds of requirements have data stewards placed on your publications, if any?
(Select ALL that apply)

	Could only publish or release a **LIMITED DATABASE**	**PRIOR REVIEW** before submission of article	**DELAYED PUBLICATION** or **EMBARGO** requirements	**PRIOR APPROVAL** before submission of article	**CO-AUTHORSHIP** required	**CITATION** required
CONSORTIUM	☐	☐	☐	☐	☐	☐
GOVERNMENT	☐	☐	☐	☐	☐	☐
PRIVATE	☐	☐	☐	☐	☐	☐

Are there any additional requirements data stewards have placed on publication?

- o CONSORTIUM ____
- o GOVERNMENT ____
- o PRIVATE ____

Do you currently face any obstacles to your database research? (Select ALL that apply)

- ☐ The kind of data I want **DO NOT or RARELY EXIST** in databases
- ☐ The kind of data I want are **NOT ACCESSIBLE** to me, although they do exist in databases
- ☐ I **CANNOT FUND** the research time necessary for analyses
- ☐ I cannot hire **RESEARCH SUPPORT**, even if I could fund them
- ☐ My institution does not, or does not always, provide **ADMINISTRATIVE INFRASTRUCTURE** (e.g., contract negotiations)
- ☐ My institution does not, or does not always, provide **RESEARCH INFRASTRUCTURE** (e.g., data analysis, data management)
- ☐ The kind of data I want are not collected with the **NECESSARY RESEARCH CONSENT** for my uses
- ☐ Something else (please describe below): ____
- ☐ ⊗*- There are no obstacles to my research -*

© 2024 Jaffe K et al. *JAMA Network Open.*

With which ancestral populations do you know you've done research with, now or in the past? (Select ALL that apply)

- ▢ African
- ▢ American Indian or Alaska Native
- ▢ Arab or Middle Eastern
- ▢ Asian
- ▢ European
- ▢ Hispanic or Latin American
- ▢ Mixed ancestries
- ▢ Native Hawaiian or Pacific Islander
- ▢ Other Indigenous Populations
- ▢ Other (please specify):
- ▢ ⊗- *I do not know/information not available –*

In your experience, have CONSORTIUM managed databases generally had an adequate sample from these ancestral populations?(Select ONE per row)

	Adequate sample	Inadequate sample	Unsure
African	o	o	o
American Indian or Alaska Native	o	o	o
Arab or Middle Eastern	o	o	o
Asian	o	o	o
European	o	o	o
Hispanic or Latin American	o	o	o
Mixed ancestries	o	o	o
Native Hawaiian or Pacific Islander	o	o	o
Other Indigenous Populations	o	o	o
Other:	o	o	o

© 2024 Jaffe K et al. *JAMA Network Open.*

In your experience, have PRIVATELY managed databases generally had an adequate sample from these ancestral populations?(Select ONE per row)

	Adequate sample	Inadequate sample	Unsure
African	o	o	o
American Indian or Alaska Native	o	o	o
Arab or Middle Eastern	o	o	o
Asian	o	o	o
European	o	o	o
Hispanic or Latin American	o	o	o
Mixed ancestries	o	o	o
Native Hawaiian or Pacific Islander	o	o	o
Other Indigenous Populations	o	o	o
Other:	o	o	o

You indicated you have NOT done research with the following ancestral populations.

With which ancestral population, if any, would you have an INTEREST in doing research if you could? (Select ALL that apply)

- ☐ African
- ☐ American Indian or Alaska Native
- ☐ Arab or Middle Eastern
- ☐ Asian
- ☐ European
- ☐ Hispanic or Latin American
- ☐ Mixed ancestries
- ☐ Native Hawaiian or Pacific Islander
- ☐ Other Indigenous Populations
- ☐ ⊗- *None of the above –*

© 2024 Jaffe K et al. *JAMA Network Open.*

What would increase the probability that you would be able to incorporate people from those ancestries into your research? (Select ALL that apply)

- ☐ Increasing the ancestral **DIVERSITY** of existing databases
- ☐ Increasing **ACCESS** to ancestrally diverse databases
- ☐ Additional **METHODS** development to support research in additional populations
- ☐ Additional **FUNDING** opportunities
- ☐ Additional **PUBLICATION** opportunities
- ☐ Additional **DEMOGRAPHIC DATA** being included in the database (e.g., ancestry, sex)
- ☐ Something else (please specify):
- ☐ ⊗- *Nothing -*

Has any of your research with PRIVATELY held data been funded in whole or in part by the National Institutes of Health (NIH)? (Select ONE)

- o Yes
- o No

What was the NIH funding used for? (Select ALL that apply)

- ☐ Data access
- ☐ Data analysis
- ☐ Data harmonization/cleaning
- ☐ Data management
- ☐ Personnel effort
- ☐ Primary data collection
- ☐ Something else (please describe):

Were those data deposited, or will they be deposited, into a "Repository for Data Resulting from NIH-Supported Research" (e.g., dbGaP) or made available through an NIH-approved alternative data sharing platform? (Select ONE)

- o Yes
- o No
- o Not sure

Why not? (Please describe)

You indicated you have NOT used ${lm://Field/2} databases.

Would you be interested in using this type of database? (Select ONE)

- o Yes
- o No

You indicated you WOULD be interested in using ${lm://Field/2} databases.

© 2024 Jaffe K et al. *JAMA Network Open.*

Why have you not used them in the past? (Select ALL that apply)

- ☐ The data are **NOT ACCESSIBLE** to me (e.g., I cannot afford it, put the right kinds of legal agreements in place, or get access in a timely manner)
- ☐ The data **DO NOT HAVE THE SPECIFIC FEATURES** that I need (e.g., size of database, the right kind of sample or phenotype, inclusion of historically under-represented ancestries)
- ☐ The data **DO NOT HAVE THE KINDS OF MANAGEMENT SUPPORT OR TOOLS** that I need (e.g., data quality or integrity, harmonization with other necessary data, or storage requirements that are not burdensome)
- ☐ **I DO NOT KNOW ENOUGH ABOUT THEM**
- ☐ Other (please describe below):

You indicated you would NOT be interested in using ${lm://Field/2} databases.

Why not? (Select ALL that apply)

- ☐ The data are **NOT ACCESSIBLE** to me (e.g., I cannot afford it, put the right kinds of legal agreements in place, or get access in a timely manner)
- ☐ The data **DO NOT HAVE THE SPECIFIC FEATURES** that I need (e.g., size of database, the right kind of sample or phenotype, inclusion of historically under-represented ancestries)
- ☐ The data **DO NOT HAVE THE KINDS OF MANAGEMENT SUPPORT OR TOOLS** that I need (e.g., data quality or integrity, harmonization with other necessary data, or storage requirements that are not burdensome)
- ☐ **I DO NOT KNOW ENOUGH ABOUT THEM**
- ☐ Other (please describe below):

How often do you have a choice between different existing genetic databases for your research?(Select ONE)

- o Always
- o Sometimes
- o Never

© 2024 Jaffe K et al. *JAMA Network Open.*

You indicated that you sometimes or never have a choice between different genetic databases for your research.

Why not? (Select ALL that apply)

- ☐ Sometimes **I DO NOT HAVE ACCESS** to a different kind of database (e.g., I do not have the right kind of familiarity with the data steward, I cannot gain timely access to the data, it costs too much)
- ☐ Sometimes a different databases **DO NOT OFFER THE DATA FEATURES** that I need (e.g., the database is not large enough; does not have the right kind of sample, phenotype, or ancestral diversity)
- ☐ Sometimes different databases **DO NOT OFFER THE DATA MANAGEMENT** that I need (e.g., lack of data quality or integrity, harmonization with other necessary data, storage requirements)
- ☐ Sometimes I am **REQUIRED TO USE** a certain database (e.g., by my employer, institution, or funder)
- ☐ Sometimes **THERE IS ONLY ONE KIND** of database available that I can use for my work
- ☐ Other (please describe below): ____

You indicated that you sometimes or always have more than one option for kind of genetic database for your research.

How would you rank these from the MOST (1) to LEAST (3) important when selecting a database for your work?

______ EASE OF ACCESS TO THE DATA (e.g., familiarity with the data-holder, efficiency in getting the data, or data access is affordable)

______ DATA FEATURES (e.g., size of database, includes the right kind of sample or phenotype, inclusion of under-represented ancestries)

______ MANAGEMENT FEATURES (e.g., data quality or integrity, harmonization with other necessary data, or storage requirements that are not burdensome)

You indicated EASE OF ACCESS is the most important factor you consider when choosing between genetic databases.

How would you rank these components of EASE OF ACCESS from the MOST (1) to LEAST (3) important?

______ **FAMILIARITY** with the data steward

______ **EFFICIENCY** in accessing the data

______ **AFFORDABILITY** of the data access/management

© 2024 Jaffe K et al. *JAMA Network Open.*

You indicated that DATABASE FEATURES are the most important factor you consider when choosing between genetic databases.

How would you rank these components of DATABASE FEATURES from the MOST (1) to LEAST (5) important?
______ **SIZE** of the database
______ The right kind of data **SAMPLING** (e.g., genotyping, whole exome sequencing)
______ Necessary **PHENOTYPES** (e.g., clinical information)
______ **HISTORICALLY UNDER-REPRESENTED ANCESTRIES** are included
______ Inclusion of comprehensive **DEMOGRAPHIC OR ANCESTRAL** information

You indicated that DATA MANAGEMENT features are the most important factor you consider when choosing between genetic databases.

How would you rank these components of DATA MANAGEMENT from the MOST (1) to LEAST (3) important?
______ Data **QUALITY OR INTEGRITY**
______ Ability to **HARMONIZE** with other necessary data
______ Ability to **STORE AND ANALYZE** data appropriately

Comparing CONSORTIUM to PRIVATE databases, which do you believe offers better...(Select ONE per row)

	**CONSORTIUM** databases better	**NEITHER**	**PRIVATE** databases better	**UNSURE**
**Ease of access**	o	o	o	o
**Database features**	o	o	o	o
**Management features**	o	o	o	o

© 2024 Jaffe K et al. *JAMA Network Open.*

Comparing GOVERNMENT (which allows individual-level access) to CONSORTIUM databases, which do you believe offers better...(Select ONE per row)

	**GOVERNMENT** databases better	**NEITHER**	**CONSORTIUM** databases better	**UNSURE**
**Ease of access**	o	o	o	o
**Database features**	o	o	o	o
**Management features**	o	o	o	o

Comparing PRIVATE to GOVERNMENT databases, which do you believe offers better... (Select ONE per row)

	**PRIVATE** databases better	**NEITHER**	**GOVERNMENT** databases better	**UNSURE**
**Ease of access**	o	o	o	o
**Database features**	o	o	o	o
**Management features**	o	o	o	o

The National Institutes of Health (NIH) are implementing broad new datasharing requirements for funded research starting in January 2023. The new policy will require funded researchers to share much of the data they use with government-managed databases.

What do you see as the potential benefits of increased genetic datasharing? (Select ALL that apply)

- ▢ **VALIDATION** of research results is enabled
- ▢ **ACCESS** to existing databases is provided
- ▢ **RE-USE** of data is promoted
- ▢ **DEMOGRAPHIC DIVERSITY** of available data may be increased
- ▢ Other (please describe below):
- ▢ ⊗- *No benefits –*
- ▢

Which of the following factors do you believe would ENCOURAGE researchers to share their data? (Select ALL that apply)

- ▢ **ACADEMIC CREDIT** for datasharing
- ▢ **ADMINISTRATIVE SUPPORT** for datasharing
- ▢ **ADDITIONAL FUNDING** to pay for time spent datasharing
- ▢ **ENFORCEMENT** of mandates for data **SHARING**
- ▢ **ENFORCEMENT** of mandates for data **QUALITY**
- ▢ **PUBLICATION PRIORITY/EMBARGO** before other researchers are able to publish with contributed data

© 2024 Jaffe K et al. *JAMA Network Open.*

- ▢ Easy **COMPLIANCE REPORTING** for data not meeting requirements
- ▢ Other (please describe below):
- ▢ ⊗- *None -*

What do you see as CHALLENGES of sharing YOUR or your TEAM'S genetic data in government databases? (Select ALL that apply)

- ▢ **TIME** required to organize, clean, and share data
- ▢ **COST** of effort required to organize, clean, and share data
- ▢ **DATA USE AGREEMENTS** that limit secondary datasharing
- ▢ **OTHERS USING YOUR DATA** to your detriment (e.g., getting "scooped" or freeloader "data parasites")
- ▢ **MISUSE** of my data (e.g., privacy concerns for participants or use of data in violation of terms)
- ▢ Other (please describe below):
- ▢ ⊗- *I don't share my own data -*
- ▢ ⊗- *None -*

What do you see as potential or existing CHALLENGES of using OTHERS' genetic data from government databases? (Select ALL that apply)

- ▢ Lack of data **QUALITY** (e.g., does not explain or adhere to best practice methods)
- ▢ **NON-COMPREHENSIVE** (e.g., does not include the kind of information necessary to conduct robust secondary analyses, for example, ancestry or sex)
- ▢ **BAD ACTORS** (e.g., who "sit" on data until outdated, intentionally do not share enough data, or scope consent such that they do not 'have' to share)
- ▢ Not being able to **PROCESS** others' data (e.g., Variant Call Format data)
- ▢ Other (please describe below):
- ▢ ⊗- *None -*

Demographics

What is your gender? (Select ONE)

- o Female
- o Male
- o Neither of these describe me, this is how I describe myself:

____

What is your race? (Select ONE)

- o American Indian or Native Alaskan
- o Asian
- o Black or African American
- o Multi-race or other
- o Native Hawaiian or Other Pacific Islander
- o White
- o None of these describe me, this is how I describe myself:

____

© 2024 Jaffe K et al. *JAMA Network Open.*

Are you Hispanic or Latino? (Select ONE)

- o Yes
- o No

Do you have another ethnicity that you'd like to report?

How do you define your seniority level? (Select ONE)

- o Trainee or student
- o Junior
- o Mid-level
- o Senior

What is the breakdown of your current support (e.g. salary)? (Total must equal 100%)
Soft Funding (i.e. grants and contracts from external institutions) : _______
Hard Funding (i.e. ongoing institutional support) : _______
Something else/Not sure : _______
Total : ________

Is there anything else that you would like to share with us?

Thank you so much for completing this survey!

© 2024 Jaffe K et al. *JAMA Network Open.*
