# Supplementary material for: Genetic Researchers’ Use of and Interest in Research With Diverse Ancestral Groups
Source: JAMA Netw Open. 2024 Apr 16;7(4):e246805. doi: 10.1001/jamanetworkopen.2024.6805 (PMC11022111; doi:10.1001/jamanetworkopen.2024.6805)
Supplement: Supplement 2. — Data Sharing Statement [file jamanetwopen-e246805-s002.pdf]

Data Sharing Statement

Jaffe. Genetic Researchers' Use and Interest in Research With Diverse Ancestral Groups. *JAMA Netw Open*. Published April 16, 2024. doi:10.1001/jamanetworkopen.2024.6805

Data
Data available: Yes
Data types: Deidentified participant data
How to access data: By request to corresponding author kaytesb@med.umich.edu
When available: With publication

Supporting Documents
Document types: None

Additional Information
Who can access the data: The data will be made available for research purposes
Types of analyses: No restriction on type of analysis
Mechanisms of data availability: Without investigator support
